# Developing Healthier Meat Products: Application of Natural Polyphenols to Reduce Hazardous Compounds During High Temperature Processing and Digestion

**DOI:** 10.3390/foods14223952

**Published:** 2025-11-18

**Authors:** Du-Xin Jin, Yu-Xuan Jin

**Affiliations:** School of Food Science and Engineering, Yangzhou University, Yangzhou 225127, China; 19895637793@163.com

**Keywords:** polyphenols, meat, polycyclic aromatic hydrocarbons, gastrointestinal digestion, meat safety, lipid oxidation

## Abstract

Meat products are popularized worldwide for their great flavor and high nutritional value. However, a high consumption of high-temperature processed meat has posed an adverse health implication, contributing to an imperative demand for healthier meat products. Polyphenols are a category of compounds with excellent antioxidant and antimicrobial properties. Accumulating evidence has demonstrated that polyphenols can reduce carcinogen formation, particularly heterocyclic aromatic amines (HAAs), polycyclic aromatic hydrocarbons (PAHs), and *N*-nitrosamines (NAs), during thermal processing of meat. Notably, polyphenols can mitigate lipid and protein oxidation during the gastrointestinal digestion of meat, underscoring the role of antioxidant polyphenols in enhancing meat consumption safety. To promote the application of polyphenols in mitigating hazardous compounds in meat products, this review elucidates polyphenols’ mitigation mechanisms against thermally generated carcinogens in meat products, analyzing their multilevel suppression pathways during processing and subsequent digestive transformation through gastrointestinal interfaces. Furthermore, this article proposes an encapsulation strategy for polyphenols to address their inherent low aqueous solubility and detrimental effects on sensory properties in meat products, aiming to enhance bioavailability while minimizing adverse organoleptic impacts. This review can provide new strategies for the application of polyphenols in developing healthier meat products and to indicate a feasible direction for future research.

## 1. Introduction

Meat is an important component of human diet due to its high nutritional value and protein content. To develop desirable flavors and cater to consumer preferences, various high-temperature cooking methods, such as barbecuing, frying, and grilling, are extensively employed. During these processes, meat ingredients undergo a series of chemical reactions, including the Maillard reaction and lipid oxidation. However, these reactions can also lead to the formation of hazardous compounds with potential carcinogenicity, including heterocyclic aromatic amines (HAAs) and polycyclic aromatic hydrocarbons (PAHs) [1]. Epidemiological studies have established a close correlation between processed meat consumption and the risk of chronic diseases [2]. The International Agency for Research on Cancer (IARC) working groups, after systematically evaluating extensive data from large-scale epidemiological studies (including cohort and case–control designs), concluded that processed meat consumption exhibits a causal association with colorectal cancer, while limited evidence suggests that daily consumption of red meat is associated with colorectal cancer. Consequently, processed meat has been classified as a Group 1 carcinogen (carcinogenic to humans) in the IARC monograph, and red meat as Group 2 [3]. These health concerns have spurred growing consumer demand for healthier meat products. Therefore, developing strategies to mitigate the formation of harmful compounds during high-temperature processing of meat is essential, with emphasis on the application of safe, cost-effective, natural additives that minimize adverse effects while preserving or enhancing meat quality.

Polyphenols are a class of plant secondary metabolites characterized by chemical structures containing multiple hydroxyl groups attached to aromatic rings. They are widely distributed in various plant tissues (such as fruits, leaves, peels, and stems) and are regarded as promising natural additives in the meat industry due to their cost-effectiveness, potent bioactive properties, and generally recognized safe status. Previous studies have extensively investigated the application of polyphenols derived from various plant sources—such as tea, spices, grape seed, and pomegranate peel—as natural alternatives for meat preservation [4]. The incorporation of these polyphenols into meat products has been demonstrated to effectively inhibit lipid and protein oxidation, and retard microbial spoilage [5,6]. Nevertheless, the roles of polyphenols extend beyond shelf-life extension and quality improvement. Bi et al. reported that marinating pork with green tea extract reduced formaldehyde levels in pan-fried meat through the formation of polyphenol–formaldehyde–amino acid adducts [7]. In a study by Manful et al., marinated and grilled beef and moose exhibited an 88–97% reduction in HAAs compared with unmarinated controls, which was attributed to the total phenolic content in the marinades [8]. The inhibitory capacity of polyphenols against hazardous compound formation stems from their distinctive molecular architectures, particularly the presence of phenolic hydroxyl groups on aromatic rings, which facilitate the quenching of reactive intermediates and disrupt radical chain propagation mechanisms underlying such toxicant generation [9]. The suppression of hazardous compound formation—such as HAAs, PAHs, and *N*-nitrosamines (NAs)—through the incorporation of polyphenols into meat products has been extensively studied [10]. However, it is important to note that the generation of these harmful substances remains an inherent and concurrent outcome of various meat processing methods. Most existing reviews have focused on the generation and inhibition pathways of a single harmful substance, but have overlooked the simultaneous presence of these hazardous compounds within meat matrices [11]. Moreover, the health hazards associated with excessive processed meat consumption originate from both exogenous toxicants formed during thermal processing and culinary preparation, and endogenous carcinogenic byproducts resulting from gastrointestinal transformation pathways. Hazardous compounds such as NAs and aldehydes may also be generated internally within the digestive tract [12]. However, the modulatory effects of polyphenols on harmful compound generation during postprandial gastrointestinal transformations of meat remain incompletely characterized.

To address these gaps, this paper comprehensively reviews the health implications of hazardous compounds in meat products, as well as the potential of polyphenol-based interventions to suppress the formation of these compounds during processing and upon consumption. It examines the multifaceted role of polyphenols in the development of healthier meat products, while also critically analyzing the potential challenges associated with their incorporation, particularly in processed meat. This review aims to provide a scientific basis for formulating polyphenol-fortified, healthier meat products.

## 2. Hazardous Compounds Produced in Meat Products

### 2.1. Heterocyclic Aromatic Amines

HAAs are a category of organic compounds characterized by a fused aromatic ring system containing at least one nitrogen heteroatom, which are commonly detected during processes of barbecuing, frying, roasting, and grilling. Based on different temperatures for their formation, HAAs fall into two groups (thermic HAAs and pyrolytic HAAs). Thermal HAAs, which belong to the IQ-type or polar HAA category, are primarily generated through Maillard reactions at temperatures between 100 and 300 °C. Pyrolytic HAAs, also known as non-polar HAAs, are produced from thermal decomposition of proteins/amino acids and free radical reactions at temperatures above 300 °C. Several HAAs have been designated as probable or possible carcinogens by IARC, of which 2-amino-3-methylimidazo[4,5-*f*]quinoline (IQ) is categorized as a Group 2A carcinogen, while 2-amino-3,4-dimethylimidazo[4,5-*f*]quinoline (MeIQ), 2-amino-3,8-dimethylimidazo[4,5-*f*]quinoxaline (MeIQx), and 2-amino-1-methyl-6-phenylimidazo[4,5-*b*]pyridine (PhIP), etc., are Group 2B carcinogens [13]. More than 30 HAAs have been identified in high-temperature processed meat, while PhIP, MeIQx, and 2-amino-3,4,8-trimethylimidazo[4,5-*f*]quinoxaline (4,8-DiMeIQx) are the most frequently detected. Certainly, the profile and concentration of HAAs in meat products are critically influenced by cooking techniques. For instance, pan-frying consistently generates higher HAA levels compared to other methods. A study on ground beef found that pan-frying produced up to 27.76 ng/g of HAAs, significantly more than oven-roasting, braising, or grilling [14]. This method-dependent formation is further illustrated by the detection of MeIQx, 2-amino-3,7,8-trimethylimidazo[4,5-*f*]quinoxaline (7,8-DiMeIQx), PhIP, and 2-amino-1,6-dimethyl-furo[3,2-*e*]imidazo[4,5-*b*]pyridine (IFP) exclusively in pan-fried pork and beef patties, not in their roasted counterparts [15]. This phenomenon may be attributed to the differential formation of heterogeneous HAA precursors, resulting from varying Maillard reaction extents, induced by distinct cooking parameters (temperature/time gradients) during thermal processing. Beyond cooking methods, the intrinsic properties of meat itself are key determinants. It was reported that pan-fried ground beef contained significantly higher HAA levels than pan-fried rib steak [15]. Shi et al. qualitatively and quantitatively analyzed HAAs in twenty-five roasted meats from six different animal breeds. A notable increase in β-carbolines HAAs (specifically Harman and Norharman) was observed in the roasted lamb sample. A marked elevation in 4,8-DiMeIQx and 7,8-DiMeIQx was detected in roasted beef and duck samples, respectively, along with higher levels of IQx and MeIQ in the roasted beef sample [16]. Therefore, the divergent HAA outcomes across studies can be attributed to meat type, fat content, free amino acid content, cooking techniques, and cooking conditions such as temperature, duration, and moisture. It should be noted that the complex interplay between processing methods and intrinsic meat components, potentially involving synergistic effects, remains incompletely understood. This knowledge gap hinders the prediction of HAA profiles and the development of targeted mitigation strategies. Moreover, there are no regulatory standards for maximum permitted levels of HAAs in diverse meat products.

### 2.2. Polycyclic Aromatic Hydrocarbons

PAHs are a class of organic compounds consisting of two or more fused benzene rings. To date, more than 30 PAHs have been identified in red meat [17]. The IARC categorizes the toxicity of PAHs through a weight-of-evidence approach evaluating three evidentiary pillars: (1) human epidemiological data demonstrating exposure–cancer incidence correlations, (2) experimental carcinogenicity in controlled animal models, and (3) mechanistic plausibility of carcinogenic pathways operating in humans. BaP achieves Group 1 classification (carcinogenic to humans) through conclusive evidence–sufficient epidemiological and animal evidence, as well as resolved molecular mechanisms. DahA (Group 2A, probably carcinogenic) demonstrates robust animal carcinogenicity, though epidemiological evidence remains limited. BaA, Chr, IP, and BbF (Group 2B, possibly carcinogenic) exhibit restricted animal evidence without a mechanistic consensus. Other PAHs, such as pyrene (Pyr), phenanthrene (Phe), and fluoranthene (Fla) (Group 3, not classifiable), lack sufficient carcinogenicity evidence across all three domains. This tiered classification reflects evidentiary completeness rather than potency differentiation [18]. Based on the toxicological characteristics of these compounds and the regulatory requirements, the European Food Safety Authority (EFSA) classifies these compounds into four subgroups, including PAH2, PAH4 (benzo[*a*]pyrene (BaP), chrysene (Chr), benzo[*a*]anthracene (BaA), benzo[*b*]fluoranthene (BbF)), PAH8 (PAH4 + benzo[*k*]gluoranthene (BkF), benzo[*g*, *h*, *i*]perylene (BgP), dibenzo[*a*. *h*]anthracene (DahA), and indeno[1,2,3-*c*,*d*]pyrene (IP)), and PAH16 [19]. PAHs, which are formed during the incomplete combustion of organic matter such as wood and oil, are often detected at higher levels in smoked, grilled, and roasted meat products [20]. The formation of PAHs in meat products exhibits strong temperature dependence. High-temperature cooking methods, including grilling, frying, and charcoal broiling, demonstrate significant PAH generation, whereas heat techniques maintaining temperatures below 100 °C (e.g., boiling, stewing, steaming) show negligible PAH formation. There is also significant variation in PAH concentrations among different high-temperature modalities under standardized cooking conditions. It has been demonstrated that smoked meats yield higher levels of PAHs compared to grilled meats [21]. However, previous studies have often overlooked crucial determinants of PAH profiles in cooked meats, such as fat composition and cooking fuel. The influence of fat type is demonstrated by meatballs formulated with sheep tail fat, which exhibited significantly higher PAH4 levels than those with beef fat due to a higher polyunsaturated fatty acid content [22]. In a study by Badyda et al., the highest concentration of PAHs was observed in pork grilled with charcoal briquettes (382,020.39 ng/m^3^), while the lowest was detected in samples cooked on a gas grill (1442.16 ng/m^3^) [23]. The European Union has established maximum permissible levels for certain PAHs in processed meat, specifically set at 5 μg/kg for BaP and 30 μg/kg for the sum of PAH4 [24]. Notably, there is an absence of explicit regulatory limits for other PAHs, such as those in the PAH8 group, in processed meat. Previous studies have demonstrated that exposure to PAHs above threshold levels exerts adverse health effects, including an elevated risk of gastrointestinal cancers [25].

### 2.3. N-Nitrosamines

In meat products, carcinogenic NAs are defined as *N*-nitroso compounds generated from the reaction of nitrite with secondary amines during processing steps like curing, roasting, and fermentation. There are approximately 20 volatile and non-volatile NAs identified in meat products, of which NDMA, NDEA, NPIP and NPYR, etc., are common volatile NAs with high carcinogenicity. According to classification by IARC, *N*-nitrosodimethylamine (NDMA) and *N*-nitrosodiethylamine (NDEA) are categorized as group 2A, *N*-nitrosodibutylamine (NDBA), *N*-nitrospiperidine (NPIP), *N*-nitrosodipropylamine (NDPA), and *N*-nitrosopyrolidine (NPYR) belong to Group 2B [26]. Substantial variations exist in the compositional profiles and concentrations of NAs across different meat product categories. In a prior study, NDMA, NPIP, and NPYR were reported to be the most frequently detected volatile NAs in one hundred kinds of cured meats with levels ranging from non-detected to 3.8, 2.9, and 10.8 ng/g [27]. Notably, the content of volatile NAs detected in different meat products is usually within the range of 0–10 ng/g, varying depending on meat type, the specific processing method, processing conditions, and preservation conditions [28]. Zhou et al. found that the formation of NDMA in Chinese sausages was attributed to lipid oxidation and microbiota [29]. Lipid oxidation products can be decomposed into nitrosating agents (such as N_2_O_3_) under high temperatures, which promote the formation of NAs [28]. Studies have indicated that certain microorganisms (such as *Enterobacteriaceae* and *Staphylococci*) in fermented sausages possess amino acid decarboxylase activity that contributes to the formation of NDMA [30]. International regulatory bodies exhibit significant heterogeneity in establishing maximum allowable thresholds for NAs within processed meat commodities. For example, China’s GB 2762-2022 stipulates a 3 μg/kg threshold for NDMA in cured meats, while the Canadian Food Inspection Agency’s compliance parameters specify differential limits: 10 µg/kg for NDEA, NDPA, NDMA, and NDBA, and 15 µg/kg for NPYR in cured products [26].

## 3. Toxic Effects of Carcinogens Generated in Meat Products

A high consumption of processed meat has been linked to the onset and progression of several chronic diseases, including obesity, insulin resistance, cardiovascular disease, and even cancer. Extensive evidence demonstrated that various carcinogens formed during thermal processing, such as PAHs, HAAs, and NAs, are potential causative factors. These carcinogenic compounds primarily exert their oncogenic effects through direct DNA damage and subsequent mutagenesis. Following biotransformation into reactive intermediates, they form mutagenic DNA adducts in targeted tissues, which drive carcinogenesis. Notably, studies have identified specific DNA adducts derived from dietary carcinogens such as BaP, PhIP, MeIQx, and 2-amino-9H-pyrido[2,3-*b*]indole (AαC) in colorectal tissues [31,32,33]. While these genotoxic agents induce DNA damage across multiple organs (e.g., liver and prostate), their mutagenic potential exhibits tissue-specific patterns. This specificity is determined by metabolic activation pathways, tissue distribution of parent compounds and metabolites, and proliferation rates of target tissues. Emerging evidence extends beyond genotoxic mechanisms to implicate oxidative stress, inflammatory responses, and dysregulated cellular signaling pathways in carcinogen-mediated tumorigenesis [34]. Prospective cohort studies have demonstrated that elevated meat consumption correlates with increased plasma concentrations of pro-inflammatory biomarkers, including interleukin-6 (IL-6), leptin, and C-reactive protein [35]. This inflammatory milieu may synergize with direct DNA damage mechanisms. For instance, BaP metabolism generates substantial reactive oxygen species (ROS) through hepatic biotransformation, leading to oxidative DNA lesions. Such ROS-induced damage interacts synergistically with BaP-DNA adducts, collectively amplifying genomic instability and mutation burden. Notably, BaP functions as critical signaling mediators during neoplastic progression, inducing CYP1A1 and CYP1B1 overexpression, establishing a pro-tumorigenic microenvironment [36]. However, the assessment of processed meat’s carcinogenicity faces significant methodological challenges. Current epidemiological evidence predominantly derives from observational studies, where definitive causal attribution is complicated by multifactorial confounding. Key interferents include collinear dietary patterns (e.g., low fiber/high fat intake), lifestyle behaviors (sedentary habits, smoking), and environmental exposures (chronic alcohol use), all of which may synergistically influence cancer risk trajectories.

## 4. Controlling the Formation of Hazardous Compounds in Meat Products by Polyphenols

### 4.1. Influence of Polyphenols on HAAs in Meat Products

**Figure 1:**
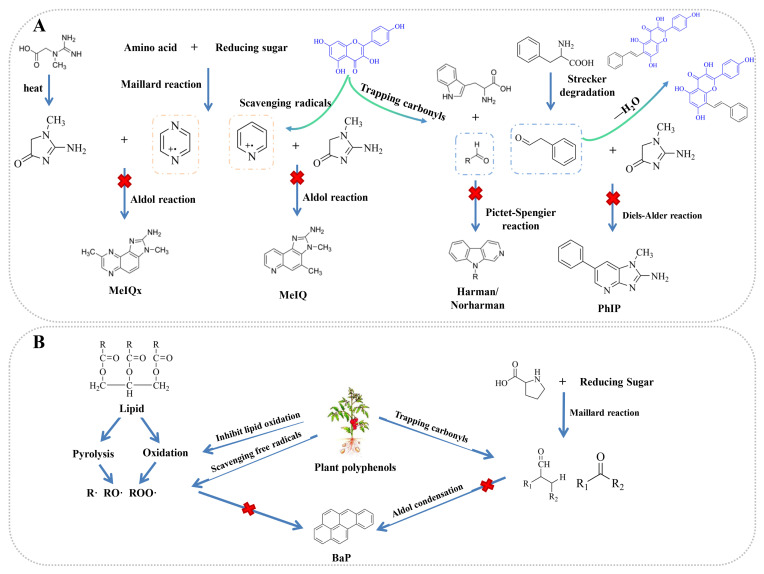
Molecular mechanisms underlying polyphenol-mediated inhibition of hazardous compounds formation. (**A**) Inhibitory pathways of kaempferol against HAA [53]; (**B**) Inhibitory pathways against PAH (represented by BaP).

Substantial research has elucidated the inhibitory capacity of polyphenols, particularly spice-derived compounds, against HAA formation in thermally processed meat. As demonstrated in Table 1, both polyphenol monomers (i.e., quercetin, naringenin, epicatechin) and complex mixtures (i.e., tea polyphenols, apple peel extract, citrus peel extract) incorporated into meat matrices significantly suppress HAA generation. Spices represent one of the most accessible and traditionally utilized natural sources of these polyphenols. For instance, Yang et al. reported that garlic, black pepper, and chili reduced total HAA contents in sausages to 584.29 ng/g, 613.11 ng/g, and 677.23 ng/g, respectively, compared to 863.86 ng/g in control samples [37]. The underlying mechanisms through which polyphenols exert their inhibitory effects primarily involve: (1) quenching free radicals, (2) inhibiting HAA precursors, and (3) interacting with reactive intermediates [17], as comprehensively reviewed in prior studies [38,39]. The radical scavenging capacity inherent to natural polyphenols constitutes a primary mechanism underlying their inhibitory effects on HAA formation. As illustrated in Figure 1A, phenolic compounds preferentially neutralize reactive radical species generated during the Maillard reaction through electron transfer from their aromatic hydroxyl groups, thereby terminating the radical propagation chains essential for HAA synthesis. Beyond antioxidant activity, polyphenols can intercept the Maillard reaction by reacting with precursors and trapping key reactive intermediates, such as dicarbonyls and Amadori rearrangement products (ARPs), consequently reducing the formation of hazardous compounds, including HAAs, acrylamide, and advanced glycation end products [40]. For example, density functional theory studied by Zhou et al. demonstrated that capsaicin inhibits β-carboline HAA formation by interacting with its intermediate, while kaempferol strongly binds phenylacetaldehyde, leading to reduced PhIP production [41]. It should be noted that some adducts formed between polyphenols and HAA precursors (i.e., 8-*C*-(*E*-phenylethenyl)-norartocarpetin) have exhibited cytotoxic effects on selective cancer cells (such as colon and liver cancer cells) [42,43]. While these adducts showed negligible genotoxicity in standardized assays, comprehensive toxicological characterization of these novel reaction products, particularly through in vitro and in vivo assessments, remains essential for safety evaluation.

However, the efficacy of polyphenols in inhibiting HAAs is not absolute and is influenced by a complex interplay of factors, leading to paradoxical effects in certain contexts. Notably, under oxidative thermal conditions, high-concentration phenolic solutions paradoxically enhance HAA formation through oxidation-derived quinones, which catalyze carbonyl group formation—a critical precursor in HAA generation [44]. For instance, in a study by Yang et al., 0.1% of ginger and Sichuan pepper promoted the formation of HAAs in cooked sausages. [37]. Ding et al. demonstrated the dual effects of several phenolics in charcoal-roasted lamb patties: rutin, quercetin, and quinic acid at 0.025 mM suppressed the formation of multiple HAAs, including IQx, 8-MeIQx, PhIP, Norharman, and Harman; however, at a higher concentration (0.625 mM), these compounds paradoxically increased HAA yields, with quercetin exhibiting the strongest promotional effect [45]. Similarly, supplementation with 0.25–0.5% tea polyphenols or (−)-epigallocatechin-3-*O*-gallate (EGCG) paradoxically elevated total HAA concentrations in roasted pork patties, specifically enhancing common HAAs (PhIP, MeIQx and 4,8-DiMeIQx) [44]. Furthermore, the inhibitory efficacy varies considerably across phenolic subclasses and structures. In a study by Wang et al. [46], *Zanthoxylum bungeanum* Maxim. leaf extract demonstrated HAA-modulating efficacy in roast beef patties through compound-specific mechanisms. The principal flavonoids, hyperoside and quercitrin suppressed PhIP formation dose-dependently, whereas the predominant phenolic acid, chlorogenic acid, increased PhIP generation in patties by up to 36.19%. This compound-specific activity was also observed by Xu et al. [47], where individual treatments with quercetin, kaempferol, and luteolin paradoxically elevated bound PhIP levels in braised duck, while their combination exhibited significant suppression. This suggests that the mitigation efficacy of polyphenol formulations may exhibit synergistic or antagonistic interactions, contingent upon their structural composition.

Substantial heterogeneity in meat processing parameters—including cooking temperature, duration, and incorporation levels—across studies further contributes to marked variability in reported efficacy. Zhang et al. observed a temperature- and time-dependent escalation in HAA concentrations within spiced pork shoulder, correlating positively with thermal processing intensity [48]. The impact of spice incorporation on HAA formation dynamics across diverse culinary and industrial processing regimes remains insufficiently elucidated, thus impeding evidence-based standardization of spice applications for carcinogen mitigation.

While polyphenols, particularly from spices, present a promising strategy for inhibiting HAA formation in thermally processed meats through multiple mechanisms, their practical application is nuanced. The observed dual effects, structural dependence, and interactions highlight that their efficacy is contingent upon concentration, specific compound structure, and processing conditions. Therefore, to determine the optimal application of polyphenol complexes in heterogeneous meat systems, further research should focus on systematic dose–effect analysis and the quantification of thermal tolerance thresholds under critical processing parameters.

**Table 1:** Effects of natural polyphenols on hazardous compounds produced during meat heat processing.

Hazardous Compounds	Polyphenols			Meat Type	Cooking or Processing Parameters	Effects	Reference
	Sources or Names	Dosage	Application Mode				
HAAs	Polyphenol-rich India session ale and white ale	A mixture consisting of 1 g oregano, 1 g parsley, 4 g mustard, 2 g salt, 8 g pepper, 1 g garlic, 25 g fresh onions, 25 mL olive oil, and 15 mL vinegar was uniformly incorporated into the unfiltered beer marinade.	Marinated the beef and moose steaks with 600 mL of each beer-based marinade at 4 °C for 12 h.	Moose and beef	Grilled at 200–250 °C for 25 min.	India session ale and wheat ale-based marination significantly reduced IQ, MeIQx, MeIQ, PhIP, Harman, and Norharman content in grilled moose and beef.	[8]
	Different spices (bay leaf, star anise, red chili) and phenolic compounds (quercetin, kaempferol, capsaicin).	Different spices (1%, 2%, 3%) and phenolic compounds (0.01%, 0.02%, 0.03%).	Different spices or phenolic compounds were individually mixed with fresh beef.	Roasted beef patties	Roasted for 10 min on each side at 200 °C.	3% red chili and 0.03% capsaicin reduced the total HAAs content by 57.09% and 68.79%, respectively.	[41]
	Green tea (GT), TP, EGCG	0.05%, 0.25% and 0.50% (*w*/*w*)	GT, TP, and EGCG powders were separately added to the ground pork uniformly.	Roasted pork patties	Roasted for 30 min (each side for 15 min) at 240 °C.	GT (0.05%, and 0.25%) and EGCG (0.05%) showed inhibition rates for MeIQx, 4,8-DiMeIQx, and PhIP ranging from 4.70 to 8.32%, 6.15–16.19%, and 5.69–24.69%, respectively.	[44]
	Chlorogenic acid, epicatechin, rutin, quercetin, and quinic acid	0.025, 0.125 and 0.625 mmol	Individually mixed with the lamb meat patties uniformly.	Charcoal-roasted lamb	Charcoal roasted at 550–600 °C for 10 min.	Chlorogenic acid and epicatechin significantly inhibited the formation of IQx, 8-MeIQx, Norharman, Harman, and PhIP.	[45]
	*Zanthoxylum bungeanum* Maxim. leaf (ZML) extract, chlorogenic acid, hyperoside, and quercitrin	For ZML extract, 0.015, 0.030 and 0.045% (*w*/*w*) chlorogenic acid (5 μg/g, 10 μg/g, 15 μg/g), hyperoside (15 μg/g, 30 μg/g, 45 μg/g), quercitrin (20 μg/g, 40 μg/g, 60 μg/g)	Mixed with the ground beef individually.	Roasted beef patties	Roasted for 10 min on each side at 225 °C in an electric oven.	ZML extract, hyperoside, and quercitrin dose-dependently inhibited the formation of PhIP. Specifically, ZML extract significantly reduced the formation of PhIP by 40–78.02%.	[46]
	Quercetin, naringenin, epicatechin, luteolin, genistein, gallic acid, resveratrol, aesculetin, and phloretin.	0.1 mmol	Individually mixed with meat slurries per phenolic compound and prepared into round patties.	Roasted lamb patties	Roasted in a 250–300 °C electric oven for 10 min.	Among these phenolic compounds, chalcone, coumarin, and stilbene compounds had higher inhibition rates, of 69.59%, 70.41%, and 72.30%, respectively. Flavonoids inhibited HAA production by 44.05–64.87%.	[49]
	Tea polyphenol (TP) powder	0.03%, 0.05%, 0.1%, 0.3% and 0.5% (*w*/*w*)	TP powder at different ratios was added into the ground meat for 4 and 6 h at 4 °C.	Grilled mutton patties	Grilled for 20 min (each side for 10 min) at 220 °C and 250 °C.	MeIQx, PhIP, and 4,8-DiMeIQx at 220 °C were reduced by 16.7%, 64.7%, and 31.1%, respectively.	[50]
	Apple peel polyphenol extract	0.1%, 0.15% and 0.3% (*w*/*w*)	Mixed or applied to the surface of beef patties	Pan-fried beef patties	Fried on each side for 10 min at 223 °C.	When applied to the surface of beef patties, the extract reduced total HAA formation by 52–71%, while when mixed internally, it inhibited HAAs by 32–45%.	[51]
	Isorhamnetin, hispidulin, cirsimaritin, and quercetin	Isorhamnetin (6 μg/g, 12 μg/g, 18 μg/g), hispidulin (3 μg/g, 6 μg/g, 9 μg/g), cirsimaritin and quercetin (1.5 μg/g, 3 μg/g, 4.5 μg/g)	Individually added into the minced meat.	Roast lamb patties	Roasted in a preheated electric oven at 220 ± 2 °C for 20 min.	Isorhamnetin and hispidulin demonstrated dose-dependent inhibition of IQ and MeIQ formation, while quercetin showed the strongest activity, with a 65.97% suppression of IQ.	[52]
	Kaempferol, naringenin, and quercetin	0.25%, 0.5%, 0.75% and 1%	Individually added to the patty.	Roasted pork patties	Roasted in an electric oven at 225 °C for 10 min per side.	Naringenin exhibited the strongest inhibitory effect on PhIP formation compared to kaempiferol and quercetin.	[53]
	Citrus peel extract	1%	Added to the patty.	Grilled pork meat patties	The patties were cooked at a temperature of 225 °C for 10 min on each side.	The extract from choline chloride-based DES significantly reduced the formation of free PhIP. MeIQx, 7,8-DiMeIQx, AαC, and norharmane, with levels of PhIP, MeIQx, and AαC decreasing by 49.2–68.3%, 34.7–53.2%, and 56.6–77.4%, respectively.	[54]
	Avocado peel extract (APE)	0.5% and 1%	Inclusion in the burgers.	Burgers	Samples were pan-fried for 6 min, with one turn at 3 min. The internal temperature reached 75 °C and the pans surface was 180/200 °C.	APE incorporation dose-dependently reduced HAAs. PhIP inhibition was lower at 0.5% than at 1% APE (67.18% vs. 88.44%), while AαC was reduced by 72.50% and 86.63% at these respective concentrations.	[55]
	Apigenin, luteolin, kaempferol, quercetin, genistein, naringenin, phlorizin, and EGCG	0.2 mM	Mixed with the ground beef individually.	Roast beef patties	The beef patties were roasted in an electric oven for 20 min (10 min per side) at 230 °C.	Phlorizin, EGCG, and quercetin effectively reduced both total HAAs and PhIP contents, with inhibition rates of 63.76% and 60.08% for phlorizin, 78.56% and 77.45% for EGCG, and 53.74% and 67.29% for quercetin.	[56]
PAHs	Heineken, Tsing Tao, Corona, Snow and Harbin beer	Marinating at a ratio of 1:1 (*w*/*v*, g/mL)	Chicken wings were marinated with different beers individually for 4 h at 4 °C.	Charcoal-grilled chicken wings	Samples were grilled 20 cm above 220 °C coals for 8 min (2 min/flip cycle), achieving 75 °C core temperature.	PAH8 generation was significantly inhibited by Heineken and Tsing Tao beer marinade, with inhibition rates of 66.92% and 31.77%, respectively.	[57]
	(−)-Epicatechin	0.2 mM/L, 1 mM/L, 5 mM/L	The beef was treated through pressure-assisted immersion using epicatechin.	Roasted beef meat cubes	Beef cubes were charcoal-roasted at 500–600 °C and a distance of 10 cm, turning every minute. After 12 min, the core temperature reached 85 ± 2.5 °C.	(−)-Epicatechin at 0.2 mM/L resulted in reduced PAH contents.	[58]
	Apple polyphenol (AP)	0.2%	The pork pieces were marinated in the prepared AP solution 1:1 (g/mL) at 4 °C for 4 h.	Barbecued pork	The meat was grilled once the charcoal surface reached 200 °C, and turned continuously until the core temperature of the meat reached 75 °C.	0.2% AP significantly inhibited PAH formation, with inhibition rates of 100% for Bap, 52.68% for PAH4, 53.10% for PAH8, and 37.36% for PAH16.	[59]
	Grape seed extract (GSE), proanthocyanidins B2 and isorhamnetin	0.01% and 0.03% GSS, 0.001% and 0.003% (*w*/*w*) of proanthocyanidin B2 and isorhamnetin	Individually incorporated into the meat during the marination process.	Bacon	The pork slices were baked at 220 °C for 6 min.	Grape seed polyphenols significantly reduced total BaP levels in bacon. At 0.01% and 0.03%, GSE decreased BaP by 57.56% and 51.16%, respectively. Proanthocyanidin B2 (0.001% and 0.003%) inhibited BaP by 49.47% and 65.43%, while isorhamnetin at these concentrations reduced it by 39.89% and 52.66%.	[60]
	Red grape pomace (RGP)	0.5%, 1%, and 3% (*w*/*w*)	The RGP was added to the burgers.	Barbecued pork burgers.	Briquettes: >300 °C (20 min preheat); Burgers: 0.22 m^2^ grill surface, 8–10 cm height, 7.5 min/side rotation, core temperature 98–101 °C.	The RGP did not significantly affect the PAH content.	[61]
	Pine needle extract (PNE) from *Cedrus deodara*	Marinade contains PNE of 0.025%, 0.05%, 0.1%, 0.2% (*w*/*w*)	All samples were marinated at 4 °C for 4 days and turned over once a day.	Smoked bacon	The marinated samples were smoked on a wire rack in a cylindrical smoker using applewood at 80 ± 5 °C for 2 h.	PNE reduced total PAH16 content in a dose-dependent manner. After PNE marinade, PAH4 was undetectable.	[62]
	Chlorogenic acid	25 mmol/L	Minced mutton was supplemented with 2 mL of chlorogenic acid solution per 20 ± 0.1 g of meat.	Roasted mutton patties	The patties were roasted at 180–200 °C smokeless electric oven for 10 min (5 min on each side) until the surface and center temperature of the patties reached 145 °C and 72 °C, respectively.	Chlorogenic acid significantly reduced Chr, BbF, BaP, and PAH4 content compared with control group.	[63]
NAs	TP, EGCG, and their palmitic acid-modified derivatives palmitoyl-TP (pTP) and palmitoyl-EGCG (pEGCG)	0.05% (*w*/*w*)	Mixed with the sausage ingredients individually.	Chinese sausages	Lean pork and pork back fat at a 4:1 ratio were mixed with ingredients. Then, the mixtures were stuffed into hog casings after curing, and subject to fermentation and drying.	TP, EGCG, pTP and pEGCG significantly inhibited the accumulation of NDMA in sausages, with a bioactivity order of EGCG > TP > pEGCG > pTP.	[29]
	Gallic acid	0.05% (*w*/*w*)	Incorporated into meat matrices.	Chinese fermented sausages	Lean pork and pork back fat at a 4:1 ratio were mixed with salt before adding NaNO_2_ and GA alone or together. After curing, the samples were stuffed, fermented and dried.	GA at 0.05% (*w*/*w*) effectively inhibited the formation of NDMA.	[64]
	*Prunus mume* polyphenol (PMP)	0.3, 0.6 and 0.9 g/kg	Mixed with sausage ingredients.	Cantonese sausage	Sausages with the addition of NaNO_2_ at 150 mg/kg meat as NIT group, and added with NaNO_2_+PMP as the PMP group. The obtained meat batter was stuffed into cellulose casings, ligated, and dried at 50 °C for 36 h until the moisture reached 18–20%.	PMP at 0.6 and 0.9 g/kg effectively reduced the NDPA and total NAs contents in sausages after 21-day storage.	[65]
	TP, AP, and cinnamon polyphenol (CP)	100, 300, and 500 mg/kg	Pork belly pieces were marinated in the brines containing polyphenols of different concentrations for 20 h at 4 °C.	Dry-fried bacon	The bellies were heat-dried at 50 °C for 1 h with 10% relative humidity before being subjected to smoking at 55 °C for 3 h with 50% relative humidity in a smoking chamber.	TP and CP at high concentrations (500 mg/kg) significantly inhibited the content of NMPhA by 38.87% and 23.09%, respectively.	[66]
	Barberry extract (BE)	200, 300 and 400 mg/kg	Added to the meat mixture.	Fermented sausages	The minced beef meat and fat were mixed with curing agents, spices, and starter culture. Then, the mixture was subject to fermentation and drying.	BE incorporation significantly reduced NPIP and NPYR levels, particularly at higher concentrations (300 mg/kg and 400 mg/kg).	[67]
	Rosemary extract, GSE, and green tea polyphenol (GTP)	0.1, 0.2, 0.3, 0.4, and 0.5 µg/g	Individually added into the prepared sausage mixture.	Western-style smoked sausage	The extracts were individually added to the prepared sausage mix. Then, the mixture was cured at 4 °C for 48 h, steamed at 80 °C for 50 min, and smoked at 75 °C for 45 min.	The extracts dose-dependently inhibited the formation of NDMA, NDPA, and NMOR in sausages.	[68]
	Catechin liposomes (CTL)	600 mg/kg	CT/CTL was evenly smeared on the samples and ripened at 22 °C at 75–80% relative humidity for 6 days.	Traditional Chinese bacon	Pork was cured at 4 °C for 48 h, baked at 60 °C for another 2 days, and surface-smeared with 600 mg/kg CT/CTL for 6 days.	The CTL achieved 40.45% NA reduction vs. 15.13% in CT-treated groups at storage terminus (49 days).	[69]
	TP	300 mg/kg	TP was added to the sausage formulations.	Cured sausage	The fresh meat batter (85%) and backfat batter (15%) were mixed together before adding TP as the control group, 150 mg/kg NaNO_2_ as the NaNO_2_ group, and TP+NaNO_2_ as the TP group.	TP significantly reduced the content of total *N*-nitrosamines in cured sausages than treated only with NaNO_2_.	[70]

### 4.2. Inhibition of PAH Formation by Polyphenols

Polyphenol-rich ingredients, including various spices, grape pomace, lignin, and rosemary extracts, have been applied to inhibit the formation of hazardous PAHs in thermally processed meat [71,72]. As summarized in Table 1, application of polyphenol monomers or mixtures, either through immediate addition or marination protocols, significantly inhibits PAH formation in processed meat, while the inhibitory effects exhibit significant variations across studies, primarily due to differences in raw meat, various polyphenols and their concentrations, as well as processing parameters (e.g., cooking temperature, heat source distance, and duration). However, current research predominantly focuses on polyphenol efficacy in homogeneous meat matrices, whereas standardized evaluation protocols for heterogeneous systems remain underdeveloped, which require a multi-matrix validation to establish universal application thresholds. Notably, PAHs generated on the meat surface may be solubilized in rendered fat during thermal processing, which subsequently permeates through intramuscular pathways (e.g., muscle fiber interfaces) and interstitial spaces within connective tissues. The direct incorporation of polyphenols into meat matrices is often hindered by compatibility challenges arising from the hierarchical organization of muscle tissue, which establishes diffusion-limiting structural barriers. Hui et al. developed a variable pressure-assisted process (−70 to −80 kPa, and pulsation ratio of 1.5) to improve the efficacy of epicatechin in inhibiting PAH formation [58]. Yang et al. developed a synergistic treatment combining cold plasma activation (40 W, 15 min, 30 L/min gas flow) with chlorogenic acid pretreatment, which exhibited greater PAH4 reduction in roasted meat compared to chlorogenic acid alone [63]. However, current research on polyphenol-functionalized meat matrices for bioactive delivery applications has not yet established standardized protocols, necessitating comprehensive studies to optimize loading efficiency.

It should be noted that polyphenols at high concentrations may not reduce PAH content. Hui et al. found that epicatechin at more than 1 mM displayed a reduced inhibitory effect against the formation of PAHs in roasted beef [58]. Previous evidence has demonstrated that the hydrophobic groups in polyphenols are prone to aggregate and promote the formation of colloidal solutions, thereby slowing down the binding rate of polyphenols with precursors responsible for the formation of PAHs, resulting in reduced inhibition of PAHs. Moreover, different phenolic compounds at high concentrations might exert antagonistic action. Thus, it is necessary to determine an appropriate concentration of polyphenols to effectively inhibit PAHs in high-temperature processed meat products.

The inhibition of PAHs by polyphenols involves a series of complex reactions. Multiple components, including protein and lipid in meat, undergo complicated reactions under high-temperature processing, which give rise to a large number of radicals. Active carbonyls from lipid oxidation can provide reaction precursors for the formation of carcinogenic PAHs [73]. The inhibitory effects of polyphenols on PAH formation are primarily mediated through their antioxidant activity (Figure 1B). Cordeiro et al. demonstrated a positive correlation between total phenolic content, antioxidant activity of vinegars, and inhibition of PAH formation using regression models [74]. AP (0.2%) significantly reduced BaP and PAH (PAH4, PAH8, and PAH16) content in barbecued pork. Notably, PAH16 levels exhibited strong positive correlations with lipid oxidation markers (TBARS and POV), suggesting that the PAH-inhibitory effect of AP may be mediated through attenuation of lipid peroxidation [59]. The hydroxyl groups on the aromatic rings of polyphenols function as potent scavengers of critical radical species (R•, RO•, ROO•) generated during thermally induced lipid oxidation, effectively intercepting PAH-precursor radicals through hydrogen atom donation [61]. Meanwhile, polyphenols can chelate transition metal ions, intercepting the production of hydroxyl radicals from the Fenton reaction.

Moreover, polyphenols exert an inhibitory effect against PAH formation by interacting with PAH precursors. The hydroxyl groups of polyphenols enable covalent and/or non-covalent interactions with lipid-derived PAH precursors (e.g., unsaturated fatty acids), generating stabilized complexes that effectively block their participation in thermal pyrolysis reactions [58]. Various chemical reactions, including the Maillard reaction and Aldol condensation, occur simultaneously during high-temperature meat cooking. Lv et al. demonstrated that the addition of glucose and several amino acids (including tryptophan, serine, cysteine, glutamine, phenylalanine, and tyrosine) significantly promoted the formation of PAHs in roasted beef patties [18]. Emerging evidence suggests that the Maillard reaction involving amino acids (such as proline) can provide hydroxymethylfurfural (HMF) as precursors for the formation of PAHs such as BaP [73]. In a study by Zhang et al., 0.01% grape seed extract significantly reduced BaP content in bacon from 1.75 μg/kg to 0.73 μg/kg, while the predominant phenolic compounds in grape seed extract including proanthocyanidin B2 and isorhamnetin significantly suppressed BaP and HMF levels in the Maillard reaction using glucose and lysine as substrates [60], indicating that phenolic compounds can inhibit BaP level by reducing the amount of precursors that participate in PAH formation. Additionally, polyphenols effectively suppress PAH formation through carbonyl compound sequestration, thereby intercepting critical pathways in both Maillard reaction cascades and thermal decomposition processes. However, current research has predominantly overlooked the dynamic changes in phenolic content during thermal processing and their interactions with Maillard reaction intermediates. Overall, the inhibitory mechanisms of polyphenols on PAH formation remain underexplored, particularly regarding specific reaction pathways. Future investigations should focus on elucidating structure–activity relationships and characterizing the resultant reaction products formed between polyphenols and PAH precursors to establish comprehensive mechanistic insights.

### 4.3. Inhibition of Polyphenols Against the Formation of NAs

Despite significant heterogeneity in polyphenol formulations and dosages across studies, these bioactive compounds consistently demonstrate notable inhibitory effects against NA formation in meat products (Table 1). For instance, Cheng et al. reported that the addition of 0.6 g/kg of *Prunus mume* polyphenols significantly reduced total NA content in Cantonese sausages by 6.67% after three weeks of storage [65]. Polyphenols exert multimodal inhibitory effects through distinct or synergistic mechanisms.

The primary mechanism involves suppressing the accumulation of NA precursors, specifically nitrite and secondary amines. Several plant polyphenols, including *Prunus mume* polyphenol, tea polyphenol, and Japanese grape powder, directly reduce residual nitrite levels in various meat products [65,70,75]. Polyphenols and secondary amines exhibit competitive binding kinetics with nitrite through parallel reaction pathways. This substrate competition fundamentally determines NA inhibitory efficiency, as the relative reaction rates between nitrite–polyphenol and nitrite–amine interactions govern precursor availability for nitrosation. A comparative kinetic analysis by Deng et al. revealed that three phenolic compounds (EGCG, epigallocatechin, and gallic acid) exhibit significantly faster nitrosation rates with nitrite compared to secondary amines [76]. This kinetic predominance establishes polyphenols as competitive nitrite scavengers, effectively blocking amine participation in nitrosation through substrate exclusion mechanisms.

Concurrently, polyphenols reduce the formation of secondary amines. As NAs in processed meat are closely correlated with biogenic amines (BAs)—precursors for NA formation—and since BA production depends on specific amino acid availability and microbial amino acid decarboxylase activity, certain polyphenols directly inhibit these decarboxylases [77]. Certain polyphenols directly inhibit these decarboxylases. For instance, specific ellagitannins reduce histamine accumulation by inhibiting histidine decarboxylase activity [78], while tea polyphenols bind to ornithine decarboxylase via hydrogen bonds and hydrophobic interactions, thereby lowering putrescine levels [79]. Given the high substrate specificity of amino acid decarboxylases, polyphenol-mediated inhibition is likewise structure-dependent. This was demonstrated in fermented sausages, where Cava lees, rich in phenolic compounds, significantly reduced tyramine and cadaverine content but exhibited no significant effect on putrescine, spermidine, or spermine [80].

Beyond precursor control, polyphenols directly block the nitrosation reaction by competitively binding to nitrosating agents or amine precursors, forming nitrosated or nitrated polyphenol derivatives. This reaction protects proteins from nitrosation and impedes NA formation. Polyphenols rapidly reduce nitrosating species (such as NO^+^, HNO_2_, N_2_O_3_) via their catechol structure to form nitric oxide (NO), thereby preventing their reaction with secondary amines. Recent evidence indicates that electrophilic sites on flavonoid A/B rings undergo nitrosation/nitration, generating nitro(so) derivatives while depleting nitrite [81]. However, comprehensive evaluation of the cytotoxicity and flavor modulation effects of these derivatives in meat products remains essential for health risk assessment and quality optimization.

Furthermore, polyphenols indirectly suppress NA formation by inhibiting protein and lipid oxidation. By providing hydrogen atoms or chelating metal ions, polyphenols interrupt lipid oxidation chain reactions and reduce secondary oxidative products such as malondialdehyde (MDA), which otherwise promote protein oxidation and increase amine production. Through structure–activity relationships, polyphenols scavenge reactive oxygen species, protecting susceptible amino acids from oxidative damage mediated by free radicals and lipid peroxidation derivatives. Moreover, they directly inhibit protein oxidation, thereby blocking the formation of secondary amines required for nitrosation. Previous studies have established a strong correlation between secondary amine content and protein oxidation, lipid peroxidation, and proteolysis [65]. Deng et al. further demonstrated that AP at 300 mg/kg significantly alleviated protein oxidation and inhibited *N*-nitroso-methyl phenylamine (NMPhA) content in dry-fried bacon [66]. Notably, high-concentration polyphenols may exhibit pro-oxidative effects, necessitating integrated multi-omics investigations combining proteomic profiling with molecular dynamics simulations to decipher the thermal processing parameters governing their functional duality.

The application of polyphenols in processed meats is limited by the instability of some phenolic compounds at high temperatures. To address this, Wu et al. [69] employed liposome encapsulation to enhance the stability and lipid solubility of catechins during meat processing and storage. The encapsulated catechins were more effective in inhibiting the formation of various NAs (e.g., NDMA, NDBA, NDPA) during long-term storage of bacon, validating the feasibility of polyphenol encapsulation in meat products. However, encapsulation efficiency critically influences the preservation efficacy of bioactive polyphenols in final products. Suboptimal encapsulation efficiency causes a substantial portion of polyphenols to remain unencapsulated, rendering them vulnerable to degradation during processing and storage. Importantly, encapsulation mechanisms differ fundamentally between hydrophilic and lipophilic polyphenols: hydrophilic variants predominantly localize in aqueous core regions, whereas lipophilic counterparts incorporate into phospholipid layers. These disparities in encapsulation mechanisms influence loading efficiency and release kinetics. Current research gaps primarily involve insufficient predictability of site-specific polyphenol release and inconsistent encapsulation performance. Future investigations should prioritize optimizing encapsulation systems to achieve site-specific release and enhanced loading efficiency of phenolic compounds in complex meat matrices, alongside quantitative evaluation of release dynamics under varied processing conditions.

## 5. Modulation of Toxicant Formation by Polyphenols During Gastrointestinal Transit of Meat Products

### 5.1. Impact of Polyphenols on Lipid Oxidation During Meat Digestion

Lipid oxidation products formed during meat digestion have been identified as critical mediators linking processed meat consumption to elevated risks of chronic diseases, particularly colorectal cancer and cardiovascular disorders, through well-established epidemiological correlations [82]. The gastrointestinal digestion of polyunsaturated fatty acids induces peroxidation cascades, generating cytotoxic aldehydes including MDA, 4-hydroxy-2-hexenal (HHE), and 4-hydroxy-2-nonenal (HNE). Cooking techniques such as grilling and roasting exacerbate this process by accelerating oxidative degradation of red meat lipids [83]. Polyphenols demonstrate concentration- and structure-dependent modulation of lipid oxidation. Experimental evidence reveals that olive polyphenols (at 300 and 600 mg/kg) significantly suppress MDA, HHE, and HNE generation during simulated digestion of high-fat beef [84]. Similarly, insoluble dietary fiber-bound polyphenols from highland barley (IDF-HBBP, 30% *w*/*w*) reduced lipid hydroperoxide (LOOH) levels in cooked pork by 77.4% during gastric digestion and 55.6% at the intestinal phase, while concurrently suppressing MDA, HHE, and HNE formation by 39.1%, 34.4%, and 37.3%, respectively, in a static in vitro gastrointestinal model [85]. Another study demonstrated that incorporating herbs/spices prior to cooking enhanced the antioxidant efficacy of beef more effectively than post-cooking supplementation did [86]. This observation confirms that polyphenols primarily act by terminating radical chain reactions rather than neutralizing pre-existing oxidation products. Therefore, incorporating polyphenol-rich compounds during food preparation becomes crucial for effectively inhibiting lipid oxidation product formation during subsequent digestion processes.

Polyphenols primarily scavenge free radicals during gastrointestinal digestion of meat products through direct interaction with hydroperoxyl (ROO•) and alkoxy (RO•) radicals by donating hydrogen atoms or electrons [84]. Paradoxically, polyphenols may enhance oxidative stress through iron redox cycling, whereby Fe^3+^ reduction to Fe^2+^ promotes hydroxyl radical (OH•) generation via the Fenton reaction [87]. Kuffa et al. reported that grape seed extract, when added at 0.1% (*w*/*w*), accelerated lipid peroxidation during simulated digestion of turkey meat, whereas it exhibited antioxidant properties at a concentration of 1% (*w*/*w*) [88]. Han et al. demonstrated that olive polyphenols at an elevated concentration (300–600 mg/kg) significantly inhibited lipid oxidation, with significant reductions in MDA, HHE, and HNE levels [84]. In a previous study, hydrophilic phenolic compounds—such as ferulic acid, chlorogenic acid, and caffeic acid—at a low dose (2.5 mg) stimulated HNE generation, while at higher concentrations (5–20 mg), these compounds significantly suppressed the production of lipid oxidation products under identical digestion conditions. However, gallic acid (2.5–20 mg) dose-dependently increased the formation of HNE in high-fat beef (4.5 g) during simulated gastrointestinal digestion [89]. Most hydrophilic polyphenols exhibited near-complete (>99%) aqueous phase partitioning in neutral water/oil systems but showed reduced hydrophilicity under simulated gastric conditions (pH 3.0). For instance, ferulic acid demonstrated 77.3% aqueous phase retention in acidic environments. Notably, gallic acid maintained near-total hydrophilicity across both pH conditions, indicating unique acid-stable partitioning behavior [90]. At low concentrations, hydrophilic polyphenols mediate Fe^3+^ reduction that enhances free radical generation, while their insufficient scavenging capacity permits reactive species (particularly •OH and HOO•) to migrate to lipid–water interfaces. These escaping radicals preferentially partition into the lipid phase due to polyphenols’ limited lipophilicity, initiating self-propagating oxidation cascades that generate cytotoxic lipid peroxidation products, including MDA and HNE. Contrarily, elevated concentrations of polyphenols exhibit antioxidant efficacy by reducing the formation of lipid oxidation products. Several hydrophilic polyphenols (e.g., caffeic acid, ferulic acid) accumulated in the lipid phase under acidic conditions can neutralize lipid radicals, while simultaneously enhancing aqueous-phase radical scavenging capacity through mass action effects. The dual-phase interception prevents radical propagation, effectively suppressing oxidative chain reactions. Therefore, the selection of appropriate phenolic constituents and concentrations is crucial for controlling lipid peroxidation in meat products during gastrointestinal digestion. Future studies should prioritize comprehensive characterization of phenolic profiles derived from plant sources (e.g., fruits, vegetables, and spices) before their application in meat processing. Notably, current understanding of gastrointestinal lipid oxidation predominantly relies on static in vitro models. Implementation of dynamic artificial digestive systems could better simulate in vivo conditions, enabling more accurate assessment of hazardous compound formation during meat digestion. This advancement would facilitate the development of mitigation strategies through optimized polyphenol incorporation to regulate chemical reactivity in meat matrices.

### 5.2. Influences of Polyphenols on Nitrosation During Meat Digestion

Extensive studies have confirmed that cured meat is associated with *N*-nitroso compound (NOC) formation during gastrointestinal digestion due to the low pH of the stomach environment [81]. As demonstrated by Bonifacie et al., nitrosylated heme iron and nitrosothiols were detected after the in vitro digestion of cooked and cured pork, irrespective of nitrite addition [91]. Accumulating evidence has demonstrated that polyphenols are promising candidates as antinitrosating agents. Ren et al. revealed that procyanidin B2, catechin, and gallic acid inhibited dimethylamine nitrosation in simulated gastric conditions in a concentration-dependent manner, with 0.2% (*w*/*w*) demonstrating remarkable efficacy (>80% inhibition) during 30 min reactions containing 2 mM NaNO_2_ [92]. In a study by Herraiz et al., the anti-nitrosative potential of catechin and quercetin was evaluated in simulated gastric environments using xanthine as a biomarker to quantify 2′-deoxyguanosine and DNA nitrosation. The compounds demonstrated potent nitrosation inhibition, with catechin (IC_50_ value of 58 μM) exhibiting greater efficacy than quercetin (IC_50_ of 101 μM) [93]. DNA nitrosation is a significant marker of molecular damage that can induce genotoxicity. As evidenced by Deng et al., phenolic compounds—specifically, EGCG, epigallocatechin, and gallic acid—inhibited the formation of NDEA at 37 °C and pH 3.0, primarily via a free radical scavenging mechanism [76]. Certainly, limited evidence has demonstrated that phenolic compounds can inhibit the formation of carcinogenic *N*-nitrosamines during meat digestion. In the presence of nitrite, (−)-epicatechin, rutin, and quercetin significantly reduced the formation of *N*-nitroso-*N*-acetyltryptophan during gastric digestion through multiple reaction pathways, including flavonoid oxidation, C-nitration, and covalent coupling with *N*-acetyltryptophan [81]. Sirvins et al. also reported that several phenolic compounds—including caffeic acid, EGCG, (−)-epicatechin, hydroxytyrosol, rutin, and chlorogenic acid—at 1 mM significantly inhibited the *N*-nitrosation of *N*-acetyltryptophan in a cured meat model. Among these, caffeic acid, EGCG, and (−)-epicatechin reduced *N*-acetyltryptophan *N*-nitrosation by 52–70% during the initial stage of gastric digestion and by 89–94% after the final stage of gastric digestion [94]. Another study adopted aqueous and emulsified models composed of various compounds to simulate complex chemical reactivity occurring during meat digestion, whereas various phenolic compounds, including chlorogenic acid and naringenin increased *N*-nitrosation by 12% and 6% in emulsified model, but inhibited *N*-nitrosation by 32% and 8% in the aqueous model [12]. The inconsistency in results regarding the inhibitory or promotive effects of polyphenols on *N*-nitrosation can be attributed to the lack of specificity in the spectrophotometric method used for nitroso compounds detection. However, it should be noted that these reactions were studied in a simulated ham matrix. Thus, the results may not be directly extrapolated to the complex conditions of real meat digestion. The contents of hazardous compounds produced during gastric digestion may be underestimated. Future studies should clarify the nitrosation of polyphenol-incorporated meat products in the body through in vitro simulated digestion models, animal experiments, and randomized controlled trials.

## 6. Limitations and Future Perspectives

The standardization of natural polyphenols as functional ingredients in meat products faces substantial challenges encompassing compositional variability and regulatory ambiguities. On one hand, source-related variability in polyphenol composition arises from botanical origin, cultivation conditions (geographical/climatic variations), and post-harvest handling. Extraction methodologies further enhance the difficulties of standardization, where solvent selection (e.g., ethanol vs. supercritical CO_2_) and processing parameters (time/temperature) directly influence phenolic profiles. The inherent complexity of polyphenol mixtures—often comprising dozens of interacting compounds—introduces unpredictable synergistic/antagonistic effects that defy conventional standardization paradigms. While total phenolic content provides a simplistic metric, biomarker-based standardization remains contentious due to incomplete mechanistic understanding of key bioactive contributors. On the other hand, regulatory fragmentation exacerbates these challenges. Divergent classifications complicate multinational product labeling. Consumer skepticism toward “extract” terminologies further conflicts with clean-label trends, necessitating nuanced ingredient declarations. A harmonized framework addressing compositional variability, dose-optimization models, and transnational regulatory alignment remains imperative to unlock the full potential of polyphenols in meat applications.

The application of polyphenols to mitigate the formation of hazardous compounds during high-temperature processing and digestion of meat also faces several challenges. First, while numerous studies have investigated polyphenol concentration-dependent mitigation of harmful compound formation across meat matrices under fixed processing conditions, critical parameters (e.g., temperature, duration, equipment) in household cooking and commercial production scales substantially deviate from controlled laboratory settings. Meanwhile, the generation of multiple toxicants during different processing stages introduces significant complexity in optimizing polyphenol dosing protocols for meat matrices. Moreover, the complexity of the gastrointestinal environment adds difficulty in estimating the dynamic changes in the levels of these compounds. Future research should prioritize investigating the efficacy of polyphenols to mitigate harmful substance formation in various domestic cooking methods, while the effect of stage-specific addition of low-dose polyphenols during meat processing should also be evaluated. Second, the inhibitory effects of natural polyphenols on different classes of hazardous compounds vary considerably due to their distinct mechanisms of action. Currently, limited data are available to manufacturers regarding the chemical behavior of polyphenols during meat processing, cooking, and digestion [12]. Therefore, further research is essential to elucidate the mechanisms by which natural polyphenols modulate the adverse health effects associated with meat consumption.

The efficacy of directly incorporating polyphenols is frequently compromised by their instability and susceptibility to degradation and oxidation during processing, storage, and digestion. Existing studies rarely address how processing parameters may unexpectedly enhance structural modifications of polyphenols, specifically phenolic oxidation pathways that generate quinones capable of driving HAA formation. Meanwhile, most studies ignored the poor aqueous solubility of some polyphenols, which limits their bioavailability. Furthermore, existing research predominantly focuses on the systematic integration of polyphenols as functional additives within meat matrix formulations. The inclusion of high concentrations of polyphenols in meat products may compromise consumer acceptance, resulting from their undesirable taste and color. It is expected to adopt the conjugated form of polyphenols and other food components (such as dietary fiber) to address the issue of polyphenols’ impact on meat flavor. Dietary fiber–phenolic interactions could improve the astringency and solubility of phenolic compounds [95]. Further studies should elucidate both synergistic and antagonistic effects of polyphenol–conjugate supplementary additive combinations on hazardous compound inhibition during thermal processing of meat products.

Polyphenols can inhibit the formation of HAAs by inducing conformational alterations of meat protein. Specifically, resveratrol demonstrates an inhibitory effect on the production of bound and free HAAs by interacting with myofibrillar proteins, reducing exposure to amino acids involved in the formation of HAAs, and increasing exposure to non-precursor amino acids [96]. However, the interaction between polyphenols (such as EGCG and theaflavin) and meat protein results in decreased meat digestibility [97,98]. Dietary meat proteins resistant to pepsin-mediated proteolysis in the gastric phase undergo colonic translocation, where microbial biotransformation generates deleterious aromatic metabolites, including phenolic derivatives and p-cresol. Some metabolites are closely related to the pathogenesis of colorectal cancer and ulcerative colitis [99].

Despite the alignment of polyphenol application in meat products with consumer demand for clean-label formulations, achieving standardized large-scale commercialization remains challenging due to their adverse effects on color and flavor profiles. Even so, the application of polyphenols in meat products is promising due to their multifunctionality. Emerging evidence has demonstrated that dietary polyphenols can reduce the fat digestion in beef patties, thereby reducing excessive fat intake related to a high consumption of processed meats [100]. Innovative strategies must be adopted when applying polyphenols to meat products. Controlled-release technology offers an effective strategy to address the above issues of polyphenols in processed meat products during high-temperature processing, which achieves controlled release of polyphenols through the following approaches: (1) encapsulating polyphenols within carriers such as microcapsules, nanoparticles, or liposomes; (2) incorporating polyphenols into film-forming materials (e.g., chitosan); and (3) loading polyphenols onto degradable materials (such as polylactic acid, cellulose nanocrystals) by electrospinning technology. Currently, various polyphenols (such as quercetin, procyanidin, plant extracts, and spices) have been incorporated into edible films, and previous evidence has demonstrated that edible packaging films incorporating polyphenols can effectively extend the shelf life of fresh meat by improving lipid oxidation and inhibiting microbial growth [101]. Encapsulation can be employed to enhance the stability and targeted efficacy of polyphenols during food processing and subsequent digestion. For instance, microcapsules of polyphenols (e.g., from mulberry or grape peel) have demonstrated improved stability and bioaccessibility in various meat models, including dried pork [102,103]. However, little attention has been paid to the impact of encapsulated polyphenols on the generation of toxicants during meat processing or cooking. Recent evidence has demonstrated the efficacy of chitosan nanoparticle-methylcellulose edible coatings in minimizing toxic compound formation in deep-fried meat [104]. Meanwhile, emerging studies demonstrate that coating deep-fried beef meatball with sesamol-incorporated chitosan-based nanocomposite edible films exhibits comparable PAH4 mitigation efficacy to that of direct sesamol addition [105]. However, the time- and temperature-dependent interfacial release kinetics governing polyphenol migration in nanocomposite coatings during multistage thermal processing remain poorly resolved, creating fundamental bottlenecks in elucidating sustained toxicant suppression mechanisms within meat matrix applications. Future research should focus on evaluating the thermal stability of polyphenol-incorporated edible films under various processing regimes, while elucidating the supramolecular interactions that mediate carcinogen suppression mechanisms during multi-stage thermal processing of meat products.

## 7. Conclusions

The safety issue of meat-based foods, particularly high-temperature processed meat, has always been a matter of great concern. Polyphenols inhibit the formation of harmful compounds in meat products through multi-target mechanisms, involving scavenging free radicals, capturing reactive carbonyls, and binding to precursors. Moreover, polyphenols can block the nitrosation reaction during high-temperature meat processing and gastrointestinal digestion. However, their effects exhibit complex concentration and structure dependence. Encapsulation strategies (microencapsulation, liposome entrapment, and edible film incorporation) represent a promising approach for optimizing polyphenol functionality in meat systems. These techniques enable simultaneous (1) stabilization of phenolic compounds, (2) enhancement of bioavailability, (3) preservation of bioactive properties, and (4) minimization of organoleptic interference in meat matrices. However, the efficacy of polyphenol-based encapsulation strategies in suppressing the formation of harmful compounds across diverse meat processing and cooking parameters has not been systematically validated. Future investigations should prioritize two critical objectives: (1) mechanistic optimization: implement multi-omics methodologies to decode polyphenol interaction dynamics within meat matrices, establish structure-activity relationship databases, and develop predictive models to optimize polyphenol selection parameters (type, concentration, temporal application) for product-specific processing scenarios. (2) developing intelligent delivery systems: advance smart stimuli-responsive packaging technologies that synchronize polyphenol release with processing phase transitions (thermal/pH changes). Research must resolve kinetic challenges in nanocomposite coatings to ensure precise delivery during critical stages (Maillard reaction, digestive release), while maintaining coating integrity under multiphase thermal stresses.

## Abbreviations

The following abbreviations are used in this manuscript:

HAAs	Heterocyclic aromatic amines
IQ	2-amino-3-methylimidazo[4,5-*f*]quinolone
IQx	2-amino-3-methylimidazo[4,5-*f*]quinoxaline
IFP	2-amino-1,6-dimethyl-furo[3,2-*e*]imidazo[4,5-*b*]pyridine
PhIP	2-amino-1-methyl-6-phenyl-imidazo[4,5-*b*]pyridine
MeIQ	2-amino-3,4-dimethylimidazo[4,5-*f*]quinoline
MeIQx	2-amino-3,8-dimethylimidazo[4,5-*f*]quinoxaline
4,8-DiMeIQx	2-amino-3,4,8-trimethylimidazo[4,5-*f*]quinoxaline
7,8-DiMeIQx	2-amino-3,7,8-trimethylimidazo[4,5-*f*]quinoxaline
PAHs	Polycyclic aromatic hydrocarbons
BaP	benzo[*a*]pyrene
BaA	benz[*a*]anthracene
BbF	benzo[*b*]fluoranthene
Chr	chrysene
BkF	benzo[*k*]gluoranthene
BgP	benzo[*g*, *h*, *i*]perylene
DahA	dibenzo[*a*. *h*]anthracene
IP	indeno[1,2,3-*c*,*d*]pyrene
Pyr	pyrene
Phe	phenanthrene
Fla	fluoranthene
NAs	*N*-nitrosamines
NDMA	*N*-nitrosodimethylamine
NDEA	*N*-nitrosodiethylamine
NPIP	*N*-nitrospiperidine
NPYR	*N*-nitrosopyrolidine
MDA	Malondialdehyde
ARPs	Amadori rearrangement products
EGCG	(−)-epigallocatechin-3-*O*-gallate
TP	Tea polyphenol
GSE	Grape seed extract
